# Microalbuminuria, Kidney Function, and Daily Physical Activity

**DOI:** 10.1155/2013/248416

**Published:** 2013-12-26

**Authors:** Baris Afsar

**Affiliations:** Department of Nephrology, Konya Numune State Hospital, Ferhuniye Mahallesi Hastane Caddesi, 42690 Konya, Turkey

## Abstract

The present study was carried out to investigate independent relationship between daily physical activity, microalbuminuria, and kidney function. The demographic characteristics and laboratory parameters were recorded for all patients. The determination of daily activities was carried out by Nottingham Extended Activities of Daily Living Scale (NEADLS) which was performed for each patient in an interview. Totally 139 patients were enrolled. In the whole group NEADLS score was correlated with age (rho: −0.759, *P* < 0.0001), clinical systolic blood pressure (rho: −0.212, *P*: 0.018), blood urea nitrogen (rho: −0.516, *P* < 0.0001), creatinine (rho: −0.501, *P* < 0.0001), uric acid (rho: −0.308, *P* < 0.0001), albumin (rho: 0.382, *P* < 0.0001), total cholesterol (rho: −0.194, *P*: 0.022), LDL-cholesterol (rho: −0.230, *P*: 0.008), hemoglobin (rho: 0.256, *P*: 0.002), creatinine clearance (rho: 0.565, *P* < 0.0001), 24-hour urinary protein excretion (rho: −0.324, *P* < 0.0001), and 24-hour urinary albumin excretion (UAE) (rho: −0.483, *P* < 0.0001). The multivariate linear regression of independent factors corelated with logarithmically converted NEADLS score (as a dependent variable) has shown that age (*P* < 0.0001), presence of coronary artery disease (*P*: 0.011), hemoglobin (*P*: 0.020), 24-hour creatinine clearance (*P*: 0.004), and 24-hour urinary albumin excretion (*P* < 0.0001) were independently corelated with NEADLS score. In conclusion, both UAE and kidney function were independently associated with daily physical activity.

## 1. Introduction

Patients with chronic kidney disease (CKD) often have decreased physical fitness and activity [1]. The main causes are muscle atrophy [2], myopathy [3], inactivity [4], malnutrition [5], and lower albumin levels [6]. Besides, anemia, inflammation, and uremic acidosis also play a role [7].

It was well demonstrated that increased urinary protein and albumin excretion are well-known risk factors for cardiovascular end-organ damage [8, 9]. Emerging data suggest that greater physical activity may be associated with less albuminuria and physical activity could protect against albuminuria [10, 11]. However, the relationship between physical activity and albuminuria is not uniform. For example, in diabetic patients, physical activity is associated with lower albumin excretion, and physical activity has led to regression of albuminuria in interventional studies [12, 13]. However, in nondiabetics these associations were not observed [14, 15]. The relationship between physical activity and albumin/protein excretion become more complex by the phenomenon of postexercise proteinuria. Transient proteinuria, including albuminuria, is common after intense exercise, and the prevalence ranges from 18% to 100%, depending on the type and intensity [16, 17]. Although all these data exist, there are few studies in the literature which specifically address the level of kidney function, urinary albumin excretion, and daily physical activity. Thus the current study was aimed to investigate the relationship between daily physical activity, kidney function, and urinary albumin excretion.

## 2. Methods

The subjects for this cross-sectional investigation were patients attending to the nephrology outpatient clinic in a state hospital for the first time or who are under followup. The study was in accordance with the Declaration of Helsinki and local approval and written informed consent was obtained from all patients. The demographic characteristics including age, sex, smoking status (smoker or nonsmoker), and clinical characteristics (presence of diabetes mellitus, presence of coronary artery disease, and the medications used) were recorded. The exclusion criteria were as follows: type 1 diabetes, cerebrovascular disease, amputation history, presence of rhythm problems, uncontrolled hypertension, hyper- and hypothyroidism, chronic liver disease, regular analgesic use, symptomatic heart failure, fracture history, and a history of physical activity apart from normal daily activities in the last 24 hours. None of the patients were taking steroids and immunosuppressants during the study period.

All patients underwent routine physical examination; blood pressure (BP) measurements and 12-lead ECG and laboratory analysis. Office BP measurements were performed using a mercury sphygmomanometer. Adequately sized cuffs (standard cuff of 23 × 12 cm or a large cuff of 34 × 15 cm) according to arm circumference were placed on the nondominant arm. The first and fifth phases of Korotkoff sounds were taken as the systolic and diastolic BP, respectively. The measurements were taken after the patients had rested for 10 min in the sitting position with the arm comfortably placed at the heart level. Two measurements were taken at 5 min intervals. Each set of two measurements was averaged to give the office systolic and diastolic BPs.

Routine biochemical analysis and 24-hour urine specimens were collected to determine creatinine clearance as well as protein and albumin excretion. An information leaflet along with a urine container was given to all subjects and they also received a verbal explanation about how to collect a proper 24-hour urine sample. After excluding the first morning urine sample of the collection day, urine was collected over 24 h, which included the first urine sample of the next morning. At the end of the collection period, the urine containers were taken to the laboratory within 2–4 h. Adequacy of urine collection was determined by the predetermined standards [18]. Insulin resistance was calculated in diabetic patients by homeostasis model assessment (HOMA-INDEX) by the following formula:

(1) (HOMA-INDEX):[fasting  plasma  glucose(in  millimoles  per  liter) ×fasting  serum  insulin(in  microunits  per  milliliter)] ×(22.5)−1.

After being given an explanation measurement of daily activities using Nottingham Extended Activities of Daily Living Scale (NEADLS) which was performed for each patient in interview. The NEADLS was originally designed in the UK, and it is also one of the most popular extended activities of daily living scales in rehabilitation centers [19]. Responses to all questions are evaluated as not performed, 0 point; with help, 1 point; on my own with difficulty, 2 points; on my own easily, 3 points. For each subsection total, and with summation of all scores, final total NEADLS scores are obtained, and they range between 0 and 66 points [19].

### 2.1. Statistical Analysis

All values are expressed as mean ± standard deviation. Data were analyzed using the program SPSS 15.0 for Windows (SPSS, Inc., Chicago, IL). *P* < 0.05 was accepted as statistically significant. Correlations were analyzed by Pearson correlation coefficient *r* if the variables are distributed normally. Spearman's correlation coefficient rho was used if the variables are not distributed normally. Analysis of NEADLS score according to CKD stages was carried out by Kruskal-Wallis Test. For the post hoc analysis of groups Bonferroni corrected Mann-Whitney *U* test was performed. Stepwise multiple linear regression was performed to identify independent variables (age, gender, body mass index, presence of diabetes, presence of coronary artery disease, hemoglobin, albumin, 24-hour urinary albumin excretion (UAE), and 24-hour creatinine clearance) related to logarithmically converted NEADLS score (as a dependent variable).

## 3. Results

Initially 173 patients were enrolled. Two patients with type 1 diabetes, 3 patients with cerebrovascular disease, 1 patient with amputation, 4 patients with rhythm problems, 2 patients with uncontrolled hypertension with 1 patient with hyperthyroidism, 2 patients with hypothyroidism, 2 patients with chronic liver disease, 7 patients with regular analgesic use, 2 patients with symptomatic heart failure, 3 patients with fracture history, and 5 patients with a history of physical activity apart from normal daily activities in the last 24 hours were excluded. Thus the final population included 139 patients. The demographic characteristics of the patients were given in Table 1. Twenty-nine patients had normal renal function, 20 patients have stage 1 CKD (defined as creatinine clearance >90 mL/min/1.73 m^2^) but have proteinuria/albuminuria and/or renal structural damage, 29 patients have stage 2 CKD (defined as creatinine clearance between 60 and 89 min/1.73 m^2^), 49 patients have stage 3 CKD (defined as creatinine clearance between 30 and 59 min/1.73 m^2^), 5 patients have stage 4 CKD (defined as creatinine clearance between 15 and 29 min/1.73 m^2^), and 7 patients have stage 5 CKD (defined as creatinine <15 min/1.73 m^2^). The laboratory parameters of the patients were shown in Table 2.

In whole group NEADLS score was correlated with age (rho: −0.759, *P* < 0.0001), clinical SBP (rho: −0.212, *P*: 0.018), blood urea nitrogen (rho: −0.516, *P* < 0.0001), creatinine (rho: −0.501, *P* < 0.0001), uric acid (rho: −0.308, *P* < 0.0001), albumin (rho: 0.382, *P* < 0.0001), total cholesterol (rho: −0.194, *P*: 0.022), LDL-cholesterol (rho: −0.230, *P*: 0.008), hemoglobin (rho: 0.256, *P*: 0.002), creatinine clearance (rho: 0.565, *P* < 0.0001), 24-hour urinary protein excretion (rho: −0.324, *P* < 0.0001), and 24-hour UAE (rho: −0.483, *P* < 0.0001).

**Figure 1:**
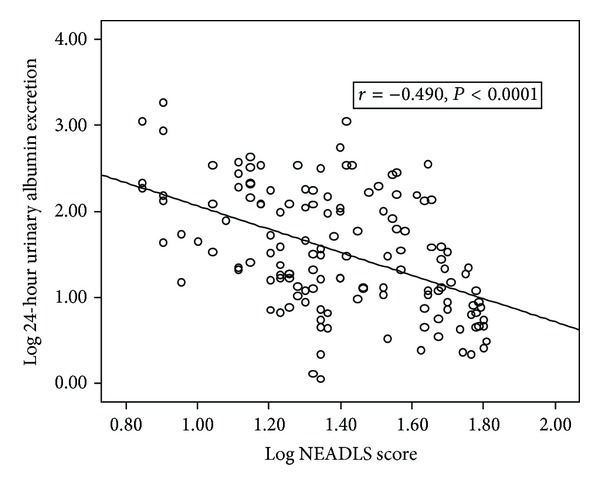
The scatter plot graph between logarithmically converted Nottingham Extended Activities of Daily Living Scale (NEADLS) score and logarithmically converted 24-hour urinary albumin excretion (UAE).

**Figure 2:**
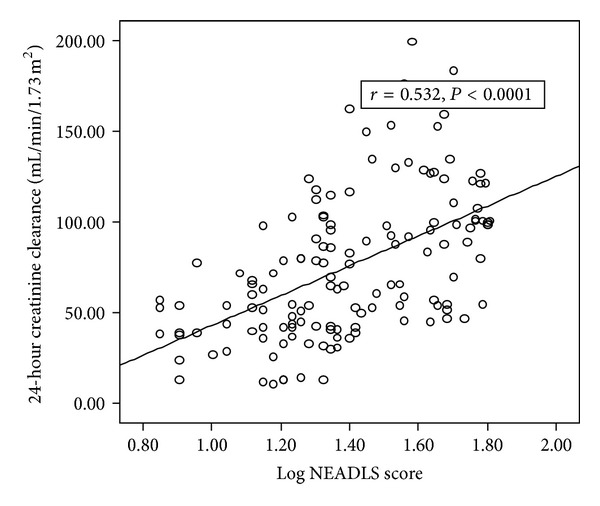
The scatter plot graph between logarithmically converted Nottingham Extended Activities of Daily Living Scale (NEADLS) score and 24 hour creatinine clearance.

Scatter plot graphic between logarithmically converted NEADLS score with logarithmically converted 24-hour urinary albumin excretion and 24-hour creatinine clearance was shown in Figures 1 and 2, respectively.

Comparison of NEADLS scores among patients with normal renal function and CKD stages were shown in Table 3.

Subgroup analysis in 57 diabetic patients has shown that NEADLS score was correlated with age (rho: −0.601, *P* < 0.0001), blood urea nitrogen (rho: −0.401, *P*: 0.002), creatinine (rho: −0.471, *P* < 0.0001), creatinine clearance (rho: 0.502, *P* < 0.0001), albumin (rho: 0.413, *P*: 0.001), 24-hour urinary protein excretion (rho: −0.438, *P*: 0.001), and 24-hour urinary albumin excretion (rho: −0.466, *P* < 0.0001). However, there was no relationship with HbA1c and insulin levels and HOMA-Index among diabetic patients. Intact parathyroid hormone (PTH) levels were available for 75 patients. In these group of patients NEADLS score was negatively associated with intact PTH levels (rho: −0.416, *P* < 0.0001).

Then multivariate linear regression of independent factors including age, gender, body mass index, presence of diabetes, presence of coronary artery disease, hemoglobin, albumin, 24-hour UAE, and 24-hour creatinine clearance related to logarithmically converted NEADLS score (as a dependent variable) was carried out. Of note 24-hour urinary protein excretion was not put into model due to high colinearity with 24-hour UAE. The results of the linear regression were shown in Table 4.

## 4. Discussion

The current study investigated the independent relationship between daily physical activity with creatinine clearance and 24-hour UAE. As a result it was demonstrated that 24-hour creatinine clearance was positively and 24-hour UAE was negatively corelated with daily activity. These findings are novel since very few studies examined the relationship between 24-hour creatinine clearance and 24-hour UAE with daily physical activity in a simultaneous way.

Recently, it was realized that both albuminuria and low glomerular filtration rate predict accelerated loss of kidney function, end-stage kidney failure, cardiovascular morbidity, and mortality [20–22]. The current study also showed that both increased UAE and decreased creatinine clearance are independently corelated with physical activity. Thus apart from cardiovascular risk, physical activity was also influenced both by UAE and creatinine clearance.

Previously, it was suggested that greater physical activity may be associated with less urinary albumin excretion. One of the mechanisms of this finding may be the endothelial dysfunction. Indeed, it was demonstrated that physical activity has protective effects on the vascular endothelium and endothelial dysfunction in the renal vasculature is associated with albuminuria [10]. Thus the relationship between increased albumin excretion and decreased physical activity may be explained in the context of endothelial dysfunction. Unfortunately, in the current study the endothelial dysfunction was not measured. Another explanation may be the presence of microangiopathy and atherosclerosis [23, 24]. Besides, increased UAE—as a measure of microangiopathy—has been suggested to influence lower limp function [25]. It has also been suggested that atherosclerosis through chronic ischemia may lead to a disturbance of bone remodeling and to a loss of the mechanical properties of the bone [26, 27] which may lead to decreased activity. Thus the association between UAE and decreased physical activity might reflect this relationship. Last but not the least, increased oxidative stress and inflammation may be potential explanations regarding the relationship between increased UAE and decreased physical activity. Indeed, it was demonstrated that physical exercise reduces both oxidative stress and inflammation [28–30], which are both related with albuminuria.

In the present study daily physical activity was positively correlated with creatinine clearance. Indeed, previous studies have shown that habitual physical activity was positively associated with renal function [31–33]. Why decrease in creatinine clearance was associated with decreased physical activity? Currently the exact mechanisms are unknown but speculations can be made. It is possible that progression of neuropathy by decreasing kidney function may contribute to a reduction in muscle mass with a consequent impact on mobility and physical activity. Another explanation may be the reduced bone mass. Although not universal, some studies have shown that subjects with mild to moderate CKD may have increased risk of osteoporosis [34]. Also other bone abnormalities seen in CKD such as osteitis fibrosa or adynamic bone disease may result in diminished physical activity. In the current study in a subgroup of patients with intact PTH levels being available, a negative association between intact PTH and NEADLS score was found. Thus more studies are needed to highlight specific relationship between PTH, bone histology, and physical activity in CKD patients.

Inflammation, acidosis, protein-energy malnutrition which are common in CKD patients may be other potential explanations given the fact that these conditions are aggravated with sedentary life and active daily life has vasculoprotective effects [7, 35, 36].

The findings of the current study may have potential implications. As an example it would be interesting to test whether drugs that decreased albumin excretion will also improve physical activity. On the other hand it should be determined whether increasing daily activity will improve kidney function.

The present study has some limitations that deserve mention. As the study was cross-sectional temporality cannot be suggested. Besides the current findings might be interpreted with caution since associations may be 2-sided. On one hand it could be possible that patients who were physically active may have lower albuminuria values and better renal function. On the other hand, that patients with a better renal function (not severe renal disease) practice regular physical activity more easily than patients with more severe renal damage. Thus cause and effect relationship cannot be suggested in such a kind of study.

Second, despite multivariate adjustment for multiple potential confounders, there remains a possibility of residual confounding. Given the fact that change in albumin excretion may occur even in the same individual, only one measurement of UAE is another limitation. Because physical activity can induce a transient increase in UAE, the association between regular physical activity and albuminuria can be difficult to study [16]. However, the patients with history of exercise apart from normal daily activity in the last 24 hours were excluded in the study. The current study also did not evaluate inflammation level and acidosis and alkalosis level.

In conclusion, daily physical activity is independently corelated with both UAE and kidney function. Studies are needed to highlight underlying mechanisms.

## Figures and Tables

**Table 1:** The demographic and clinical parameters of 139 patients.

Variable	
Age (years) (mean ± SD)	52.6 ± 15.7
Sex (male/female) (*n*:)	67/72
BMI (kg/m^2^) (mean ± SD)	28.9 ± 5.9
Type 2 diabetes (present/absent) (*n*:)	57/82
Smoking (present/absent) (*n*:)	30/109
Presence of CAD (*n*:)	18/121
Use of ACE inhibitor (present/absent) (*n*:)	27/112
Use of calcium channel blocker (CCB) (present/absent) (*n*:)	27/112
Use of *β*-blocker (present/absent) (*n*:)	25/114
Use of *α*-blocker (present/absent) (*n*:)	4/135
Use of diuretic (present/absent) (*n*:)	21/118
Use of AT2 blocker (present/absent) (*n*:)	37/102
Use of spironolactone (present/absent) (*n*:)	8/131
Office systolic blood pressure (SBP) (mmHg) (mean ± SD)	132.3 ± 16.9
Office DBP (mmHg) (mean ± SD)	81.15 ± 9.8

SD: standard deviation; BMI: body mass index; CAD: coronary artery disease; ACE: angiotensin converting enzyme; CCB: calcium channel blocker; *β*: beta-Adrenergic; *α*: alpha-adrenergic; AT2: angiotensin receptor blocker; SBP: systolic blood pressure; DBP: diastolic blood pressure.

**Table 2:** The laboratory parameters of 139 patients.

Variable	
Fasting blood glucose (mmol/L)	6.72 ± 2.70
Blood urea nitrogen (mmol/L)	8.14 ± 5.28
Creatinine (*µ*mol/L)	121.1 ± 73.4
Sodium (mmol/L)	140.3 ± 3.5
Potassium (mmol/L)	4.60 ± 0.57
Calcium (mmol/L)	2.35 ± 0.15
Phosphorus (mmol/L)	1.16 ± 0.21
Uric acid (*μ*mol/L)	342.6 ± 117.8
Albumin (g/L)	42.2 ± 4.7
Total cholesterol (mmol/L)	4.83 ± 1.15
LDL-cholesterol (mmol/L)	2.90 ± 0.87
HDL-cholesterol (mmol/L)	1.17 ± 0.29
Triglyceride (mmol/L)	1.84 ± 1.08
TSH (mU/L)	2.13 ± 1.30
Hemoglobin (g/L)	132.2 ± 16.5
Creatinine clearance (mL/min)/1.73 m^2^	75.3 ± 39.4
24-hour urinary protein excretion (mg/day)	641.1 ± 1019.1
24-hour urinary albumin excretion (mg/day)	116.0 ± 234.1

Data are mean ± SD except when stated otherwise ALT: alanine aminotransferase; AST: aspartate aminotransferase; LDL: low-density lipoprotein; HDL: high-density lipoprotein, TSH: thyroid stimulating hormone; T3: triiodothyronine; T4: thyroxine.

**Table 3:** Comparison of NEADLS scores among patients with normal renal function and CKD.

NEADLS score		*P* for trend
Normal renal function (group 1)	42.9 ± 15.8^III,IV,V,VI^	<0.0001
Stage 1 CKD (group 2)	36.3 ± 14.8^IV,V,VI^	
Stage 2 CKD (group 3)	25.9 ± 13.5^I^	
Stage 3 CKD (group 4)	23.4 ± 13.6^I,II^	
Stage 4 CKD (group 5)	13.2 ± 5.5^I,II^	
Stage 5 CKD (group 6)	15.4 ± 4.0^I,II^	

*P* value is based on Kruskal-Wallis test (groups with significant differences according to the post hoc testing are shown in superscript numbers).

**Table 4:** Results of linear regression of independent factors corelated with Nottingham Extended Activities of Daily Living Scale score.

	*β*	Beta	95% CI	*P*
Age	−0.010	−0.589	−0.013–(−0.008)	<0.0001
Being female	0.013	0.025	0.040–0.066	0.638
Presence of diabetes	0.022	0.043	−0.032–0.076	0.415
Presence of coronary artery disease	−0.101	−0.134	−0.178–(−0.024)	0.011
Body mass index	0.001	0.006	−0.004–0.005	0.910
Hemoglobin	0.020	0.128	0.003−0.037	0.020
Albumin	0.042	0.076	−0.017–0.101	0.166
24-hour creatinine clearance	0.002	0.163	0.001–0.007	0.004
24-hour urinary albumin excretion	−0.021	−304	−0.032–(−0.010)	<0.0001

*β*: Partial regression coefficient; Beta: partial correlation coefficient.

## References

[1] Brodin E, Ljungman S, Sunnerhagen KS (2008). Rising from a chair: a simple screening test for physical function in predialysis patients. *Scandinavian Journal of Urology and Nephrology*.

[2] Kouidi E, Albani M, Natsis K (1998). The effects of exercise training on muscle atrophy in haemodialysis patients. *Nephrology Dialysis Transplantation*.

[3] Diesel W, Emms M, Knight BK (1993). Morphologic features of the myopathy associated with chronic renal failure. *American Journal of Kidney Diseases*.

[4] Johansen KL, Shubert T, Doyle J, Soher B, Sakkas GK, Kent-Braun JA (2003). Muscle atrophy in patients receiving hemodialysis: effects on muscle strength, muscle quality, and physical function. *Kidney International*.

[5] Pupim LB, Flakoll PJ, Levenhagen DK, Ikizler TA (2004). Exercise augments the acute anabolic effects of intradialytic parenteral nutrition in chronic hemodialysis patients. *American Journal of Physiology: Endocrinology and Metabolism*.

[6] Johansen KL, Chertow GM, da Silva M, Carey S, Painter P (2001). Determinants of physical performance in ambulatory patients on hemodialysis. *Kidney International*.

[7] - Kosmadakis GC, John SG, Clapp EL (2012). Benefits of regular walking exercise in advanced pre-dialysis chronickidney disease. *Nephrology Dialysis Transplantation*.

[8] Abdelhafiz AH, Ahmed S, El Nahas M (2011). Microalbuminuria: marker or maker of cardiovascular disease. *Nephron Clinical Practice*.

[9] Gerstein HC, Mann JFE, Yi Q (2001). Albuminuria and risk of cardiovascular events, death, and heart failure in diabetic and nondiabetic individuals. *Journal of the American Medical Association*.

[10] Robinson ES, Fisher ND, Forman JP, Curhan GC (2010). Physical activity and albuminuria. *American Journal of Epidemiology*.

[11] Ochodnicky P, Henning RH, van Dokkum RPE, de Zeeuw D (2006). Microalbuminuria and endothelial dysfunction: emerging targets for primary prevention of end-organ damage. *Journal of Cardiovascular Pharmacology*.

[12] Calle-Pascual A-L, Martin-Alvarez P-J, Reyes C, Calle J-R (1993). Regular physical activity and reduced occurrence of microalbuminuria in type 2 diabetic patients. *Diabete et Metabolisme*.

[13] Lazarevic G, Antic S, Vlahovic P, Djordjevic V, Zvezdanovic L, Stefanovic V (2007). Effects of aerobic exercise on microalbuminuria and enzymuria in type 2 diabetic patients. *Renal Failure*.

[14] Finkelstein J, Joshi A, Hise MK (2006). Association of physical activity and renal function in subjects with and without metabolic syndrome: a review of the third National Health and Nutrition Examination Survey (NHANES III). *American Journal of Kidney Diseases*.

[15] Metcalf PA, Baker JR, Scragg RKR, Dryson E, Scott AJ, Wild CJ (1993). Albuminuria in people at least 40 years old: effect of alcohol consumption, regular exercise, and cigarette smoking. *Clinical Chemistry*.

[16] Bellinghieri G, Savica V, Santoro D (2008). Renal alterations during exercise. *Journal of Renal Nutrition*.

[17] Poortmans JR, Rampaer L, Wolfs J-C (1989). Renal protein excretion after exercise in man. *European Journal of Applied Physiology and Occupational Physiology*.

[18] Junge W, Wilke B, Halabi A, Klein G (2004). Determination of reference intervals for serum creatinine, creatinine excretion and creatinine clearance with an enzymatic and a modified Jaffé method. *Clinica Chimica Acta*.

[19] Turner-Stokes L, Turner-Stokes T (1997). The use of standardized outcome measures in rehabilitation centres in the UK. *Clinical Rehabilitation*.

[20] Go AS, Chertow GM, Fan D, McCulloch CE, Hsu C-Y (2004). Chronic kidney disease and the risks of death, cardiovascular events, and hospitalization. *The New England Journal of Medicine*.

[21] Halbesma N, Kuiken D-S, Brantsma AH (2006). Macroalbuminuria is a better risk marker than low estimated GFR to identify individuals at risk for accelerated GFR loss in population screening. *Journal of the American Society of Nephrology*.

[22] Iseki K, Kinjo K, Iseki C, Takishita S (2004). Relationship between predicted creatinine clearance and proteinuria and the risk of developing ESRD in Okinawa, Japan. *American Journal of Kidney Diseases*.

[23] Furtner M, Kiechl S, Mair A (2005). Urinary albumin excretion is independently associated with carotid and femoral artery atherosclerosis in the general population. *European Heart Journal*.

[24] Jørgensen L, Jenssen T, Johnsen SH (2007). Albuminuria as risk factor for initiation and progression of carotid atherosclerosis in non-diabetic persons: the Tromsø Study. *European Heart Journal*.

[25] Bruce DG, Davis WA, Davis TME (2005). Longitudinal predictors of reduced mobility and physical disability in patients with type 2 diabetes: the fremantle diabetes study. *Diabetes Care*.

[26] Bagger YZ, Tankó LB, Alexandersen P, Qin G, Christiansen C (2006). Radiographic measure of aorta calcification is a site-specific predictor of bone loss and fracture risk at the hip. *Journal of Internal Medicine*.

[27] Laroche M, Ludot I, Thiechart M (1995). Study of the intraosseous vessels of the femoral head in patients with fractures of the femoral neck or osteoarthritis of the hip. *Osteoporosis International*.

[28] Laufs U, Urhausen A, Werner N (2005). Running exercise of different duration and intensity: effect on endothelial progenitor cells in healthy subjects. *European Journal of Cardiovascular Prevention and Rehabilitation*.

[29] Werner C, Fürster T, Widmann T (2009). Physical exercise prevents cellular senescence in circulating leukocytes and in the vessel wall. *Circulation*.

[30] Werner C, Hanhoun M, Widmann T (2008). Effects of physical exercise on myocardial telomere-regulating proteins, survival pathways, and apoptosis. *Journal of the American College of Cardiology*.

[31] Robinson-Cohen C, Katz R, Mozaffarian D (2009). Physical activity and rapid decline in kidney function among older adults. *Archives of Internal Medicine*.

[32] Hawkins MS, Sevick MA, Richardson CR, Fried LF, Arena VC, Kriska AM (2011). Association between physical activity and kidney function: national health and nutrition examination survey. *Medicine and Science in Sports and Exercise*.

[33] Hallan S, de Mutsert R, Carlsen S, Dekker FW, Aasarød K, Holmen J (2006). Obesity, smoking, and physical inactivity as risk factors for CKD: are men more vulnerable?. *American Journal of Kidney Diseases*.

[34] Jørgensen L, Jenssen T, Ahmed L, Bjørnerem Å, Joakimsen R, Jacobsen BK (2007). Albuminuria and risk of nonvertebral fractures. *Archives of Internal Medicine*.

[35] - Pöss J, Ukena C, Mahfoud F (2011). Physical activity is inversely associated with microalbuminuria in hypertensive patients at high cardiovascular risk: data from I-SEARCH. *European Journal of Preventive Cardiology*.

[36] Kosmadakis GC, Bevington A, Smith AC (2010). Physical exercise in patients with severe kidney disease. *Nephron Clinical Practice*.

